# The Role of the Calcitonin Peptide Family in Prostate Cancer and Bone Metastasis

**DOI:** 10.1007/s40610-017-0071-9

**Published:** 2017-08-02

**Authors:** Jessica Isabel Warrington, Gareth Owain Richards, Ning Wang

**Affiliations:** The Mellanby Centre for Bone Research, Department of Oncology and Metabolism, The University of Sheffield, Beech Hill Road, Sheffield, S10 2RX UK

**Keywords:** Prostate cancer, Bone metastasis, Calcitonin peptides, Adrenomedullin, Receptor activity modifying proteins, Bone microenvironment

## Abstract

**Purpose of Review:**

This study is to highlight recent discoveries associated with the role of calcitonin peptide family and their receptors in prostate cancer progression and bone metastasis.

**Recent Findings:**

Studies have linked adrenomedullin (AM), calcitonin (CT) and calcitonin gene-related peptide (CGRP) to the spread of prostate tumours to the bone. AM can induce a metastatic phenotype in prostate cancer cells through its action on TRPV2 calcium channels and is also capable of influencing localised levels of RANKL in the bone to favour tumourigenesis. CT utilises A-kinase anchoring proteins to indirectly act on PKA and promote metastasis in prostate cancer. The receptor for CT contains a PDZ-binding domain, the deletion of which stops metastasis to the bone in orthotopic prostate models.

**Summary:**

Recent findings show strong evidence for the role of calcitonin peptides and receptors in prostate cancer and bone metastasis. Further research could provide potential prognostic markers and therapeutic targets for prostate cancer patients.

## Introduction

The calcitonin family of peptides all share structural similarities with calcitonin (CT), a hypocalcemic hormone secreted from the thyroid gland. Calcitonin gene-related peptide (CGRP) is named appropriately as it is an alternative splicing of CT gene. Amylin is another peptide in this family, sharing 40% amino acid identity with CGRP. It was first isolated from amyloid deposits in the pancreas of Type 2 diabetes patients and is co-secreted with insulin in β-islet cells. In addition to their structural similarity, amylin and CGRP also have hypocalcemic effects [1]. Another member of this peptide family is adrenomedullin (AM) which was first described in 1993 when identified in a human pheochromocytoma patient [2]. It is a potent vasodilator, acting directly on renal, cerebral and lung circulation systems as well as the vascular supply of the bone. Adrenomedullin 2 (also known as intermedin) has only recently been identified and appears to have similar physiological effects to those of AM [3].

The receptors for these peptides are unusual in that they are formed by two separate proteins, a seven-transmembrane domain G protein-coupled receptor and a receptor activity modifying protein (RAMP). The GPCR may be either the calcitonin receptor (CTR) or calcitonin-like receptor (CLR) with one of the three RAMPs (Table 1). The CTR was first cloned in 1991, with a sequence homologous to class II GPCRs, a group including peptides such as parathyroid hormone (PTH) and parathyroid hormone-related peptide (PTHrP). The CLR was then later identified with high homology to CTR. RAMP1 was discovered to co-express with CLR forming the receptor for CGRP and RAMPs 2 and 3 couple with CLR to form two distinct AM receptors (AM1 and AM2). RAMPs have been found to share 30% amino acid sequence identity, and when expressed with CTR, they form three different amylin receptors [1]. Interestingly, RAMPs 2 and 3 have also been shown to interact with PTH1 and PTH2 receptors at the cell surface [4]. In primary mouse osteoblasts, there is evidence that PTH can induce RAMP3 mRNA expression through the cAMP-PKA pathway, commonly used by GPCRs [5].

**Table 1:** Receptors for calcitonin family peptides

Ligand	Receptor	Components
Calcitonin	CTR	CTR
Calcitonin gene-related peptide	CGRP	CLR + RAMP1
Adrenomedullin	AM1	CLR + RAMP2
Adrenomedullin	AM2	CLR + RAMP3
Amylin	AMY1	CTR + RAMP1
Amylin	AMY2	CTR + RAMP2
Amylin	AMY3	CTR + RAMP3

The calcitonin family and their receptors have a wide range of physiological and pathological functions which have been widely discussed in the literature [6–9]. Due to their well-established roles in vital cellular mechanisms, it is unsurprising that they also feature as promoters of tumourigenesis across many different cancer types. For many years, the CT-CTR axis has been shown to be important in many malignant diseases. Activation of this axis stimulates invasion, angiogenesis, chemoresistance and metastasis in many cancer cell lines. Calcitonin and its receptor has been often linked in the literature with prostate cancer specifically [10–13]. Retrospective studies have found that although advanced prostate cancer can metastasize to various sites, the most common is the bone. Ninety percent of patients who die from prostate cancer have bone metastases and there is evidence that approximately 50% of those patients survive only 30–35 months after diagnosis [14].

There is currently a lack of clinically useful biomarkers that can predict bone metastasis in prostate cancer and few therapies that can prevent tumour growth in this environment. Once bone metastases have been established, prostate cancer is considered incurable. Current investigations that aim to prevent these metastases require an understanding of two key contributors: the metastasis-initiating cancer cells (the “seeds”) and the bone microenvironment which can support these cells (the “soil”) [15]. The relationship between the calcitonin peptide family and prostate cancer metastasis is a novel research topic, and it is therefore apt to appraise recent discoveries in the involvement of these peptides in prostate cancer metastasis-initiating cells and their possible roles in regulating the bone microenvironment. In this review, we will first highlight studies investigating members of the calcitonin peptide family in prostate cancer and their role in metastasis, before focusing on the influence of these peptides within the bone microenvironment.

## The Role of the Calcitonin Family in Cancer and Metastasis

The calcitonin family of peptides have been linked with cancer in many studies since their first discovery. Calcitonin and its receptor have long been implicated in prostate cancer since it was found in 1992, that the number of cells expressing CT increases with prostate cancer progression [16]. Further investigations have found that the CT-CTR axis is important in prostate cancer for growth, invasion and inducing EMT. A decade after these initial findings, studies were also able to show the role of CT and its receptor in promoting metastasis in prostate cancer [12]. Data shows that CT is secreted from the prostate epithelium and is increased sevenfold in malignant prostate tissues compared with benign. CT can promote prostate cancer cell growth via adenylyl cyclase and calcium/phospholipid pathways and can also increase invasion of cells in vitro [11].

Investigative studies of AM have been ongoing for more than 20 years and have demonstrated this multifunctional peptide to be significantly involved in the tumourigenesis in many different cancer types [6]. AM is not only upregulated in cancer cells but also promotes angiogenesis in hypoxic conditions and prevents apoptosis whilst also suppressing the immune system [17, 18]. Studies have also linked AM to the role of progressing tumours to bone metastasis; this could be anticipated when coupled with AM’s effect on bone physiology which will be detailed subsequently [19]. Unsurprisingly, AM2 has been found to be implicated in cancer progression most recently in breast and prostate; however, mechanisms remain relatively unclear. AM2 can stimulate cell migration and tube formation in angiogenesis in vitro models and is also upregulated in hypoxic conditions [20, 21]. CGRP and its receptors have also been linked with the upregulation of survival pathways. Several studies have found CGRP to increase proliferation by triggering the MAPK pathway [22]. Amylin is upregulated in pancreatic cancer and is seen to increase in diabetic patients with pancreatic cancer and continue to increase in pancreatic patients with normal glucose levels; however, its role in other cancers has not been further investigated [23].

## Prostate Cancer and Metastasis: Adrenomedullin

One of the key processes in tumour metastasis is cell migration; many components of this mechanism are regulated by intracellular calcium (Ca^2+^) levels. Ca^2+^ entry is often induced via ion channels, some of which are called “transient receptor potential” (TRP) channels. These have been reported to be implicated in tumourigenesis, and more specifically, the TRPV2 channel has been found to promote the progression of prostate cancer towards a more aggressive phenotype by stimulating migration and invasion in cells [24]. A recent study has found a role for AM in stimulating TRPV2 and indirectly promoting tumour progression and metastasis. Treatment of AM to various prostate cancer cell lines results in phosphorylation of β1 integrin and Focal Adhesion Kinase (FAK). This downstream response is consistent with the ability of AM to increase the invasive and migratory capacity of prostate cancer cells by approximately 50%. siRNA knockdown of TRPV2 in these cells abolishes all effects seen by AM treatment leading to the conclusion that this Ca^2+^ channel is regulated by AM and helps to promote prostate cancer cells to require a metastatic phenotype [25•].

A polyclonal antibody designed to block all the receptor components of AM (RAMP2, RAMP3 and CLR) was used to treat prostate cancer cells in vitro resulting in a dose-dependent reduction in proliferation and a 95% decrease in invasion. DU145, a prostate cancer cell line with moderate metastatic capability, was used in a mouse xenograft model. After anti-AM treatment, tumour weight was reduced fourfold with no metastases found. Compared with controls which showed the presence of metastasis in the lung, kidney and spleen, this shows that AM has a strong effect on prostate tumour metastasis. The blocking of AM also caused a reduction in the lymphatic vasculature of the tumours but not the surrounding tissue. This finding is very important when considering the use of AM antagonists in therapeutic intervention of prostate cancer and bone metastasis. To further investigate this, the group, cultured smooth muscle cells (SMCs) with conditioned media from lymphatic endothelial cells, resulting in SMC migration. This effect could then be reversed when blocking AM, suggesting its role in SMC recruitment may drive vessel formation in prostate cancer tumours [26]. This is an interesting role for AM which has already been implicated in angiogenesis in different cancer types [27–29] but now is found to capable of promoting tumour lymphangiogenesis.

Breast cancer is another highly aggressive and metastatic disease and there have been recent studies of AM in breast cancer metastasis which may shed light on mechanisms that are shared in prostate cancer as well. Lysyl oxidase (LOX) is an enzyme that can form bonds between collagen and elastin fibrils, to ensure extracellular matrices are formed stably. LOXL2 can contribute to tumour expression by inducing an epithelial-to-mesenchymal transition (EMT) in cells, an event that is vital for tumours to become metastatic. Recently, a link between LOXL2 and RAMP3 expression has been found in many different cancer cell lines including MDA-MB-231, a metastatic breast cancer cell line. The inhibition of LOXL2 with shRNA caused a mesenchymal-to-epithelial transition in breast cancer cells and a reduced invasive phenotype; these effects were then reversed using recombinant RAMP3. RAMP3 expression was observed to decrease with LOXL2 inhibition suggesting a regulatory role for the enzyme. RAMP3 inhibition resulted in reduced tumour development in vivo*,* with a lower tumour microvessel density compared with controls [30]. This study shows that RAMP3 may be specifically involved in many processes key for cancer metastasis and LOXL2 stimulates these actions upstream.

It is clear looking at recent discoveries that AM has a role to play in prostate cancer and bone metastasis, so it would be wise to also consider AM2 which has already been shown to have similar physiology functions. However, this peptide has only recently been investigated in cancers including colorectal, breast and prostate [31–33]. Plasma AM2 levels were shown to strongly correlate with many prognostic factors used to assess prostate cancer patients including a 5-year metastasis, Gleason’s score and tumour node metastasis [33]. Consequently, future investigations may regard AM2 as a potential key player in prostate cancer and bone metastasis.

## Prostate Cancer and Metastasis: Calcitonin

Calcitonin was shown in studies conducted almost a decade ago, that it may have a role to play in the metastasis of prostate cancer; however, not until recently, the mechanisms behind this have been investigated in depth.

It is known that many GPCRs including CTR can phosphorylate PKA, a serine threonine kinase, through a cAMP-dependent pathway. A detailed study into the interactions between CT and PKA have revealed how CT can promote an EMT in prostate cancer cells. The action of PKA can be determined by its discrete location and the duration of activation, a spatiotemporal effect. A-kinase anchoring proteins (AKAPs) provide this spatial specificity for PKA by tethering an activating enzyme in different intracellular environments. Thakkar, et al. hypothesised that CTR increases the metastatic capability of prostate cancer cells by selectively activating AKAP and indirectly influencing PKA.

Treatment with CT in PC3 cells causes an increase in invasion and adhesion which can be abolished using Ht-31Pro, an AKAP inhibitor. There are more than 50 different AKAPs, and so, to identify which one is acting in CTR-induced PKA activation, the group used siRNA to knockdown different AKAPs to see which caused an inhibitory effect. By focusing on just the AKAPs localised to the plasma membrane, they found AKAP2 can reverse CT-induced invasion of prostate cancer cells. Further evidence showed CT could cause increases in proliferation, colony formation and orthotopic tumours with bone metastasis, and all these effects could be reversed with AKAP2 knockdown. Analysis of different prostatic tissues with immunohistochemistry found that AKAP2 levels are barely detectable in benign prostatic human tissue but becomes visible in localised prostate cancer and significantly higher in metastatic prostate cancer [34•].

Many peptide receptors contain PDZ-binding motifs which are a common mechanism for cancer metastasis. Aljameeli, et al. have recently investigated the PDZ motif in CTR to see if this is an appropriate mechanism for CTR in prostate cancer metastasis. By mutating the domain in different prostate cancer cell lines, they found that it is required for tight junction stability, and that its deletion reversed a 2.5-fold increase in invasion seen in prostate cancer cells stimulated with CT. This mutation has no effect on cAMP pathway activation suggesting ulterior signaling pathways may be utilised after CTR activation. Yeast 2 hybrid complementation is a method for identifying important protein-protein interactions; the strongest signal the group found was on zona occludens 1 (ZO-1). This is novel finding showing CTR may act with ZO-1, a key protein in tight junction regulation. In vivo studies showed deletion of PDZ-binding domain on CTR produces no metastasis in the femur which were fourfold more frequent in control mice [35••]. The relationship between CT and ZO-1 could be further investigated in the future to provide additional evidence of its vital role in the promotion of prostate cancer cell metastasis. It is also interesting to note that RAMP3 contains a PDZ-binding motif, and therefore these mechanisms could be mirrored in other calcitonin peptides.

## Prostate Cancer and Metastasis: Calcitonin Gene-Related Peptide

Many different neuropeptides including CGRP are expressed in the prostate gland in both autonomic and sensory nerves and in neuroendocrine cells. Neuroendocrine cells are known to differentiate more frequently in prostate cancer compared with many other cancers and correlates with tumour progression [36]. It has long been known that CGRP can increase the invasive and migratory capacity of prostate cancer cells by 30–40% in vitro and that serum levels correlate with prostate cancer progression in human patients [37, 38]. However more recently studies associated with RAMP1 may provide evidence of the CGRP receptor promoting prostate cancer and metastasis to the bone.

NKX3.1 is a prostate specific transcription factor that functions in prostate development as well as tumour progression. NKX3.1 deletion in mice leads to formation of premalignant lesions in prostate. Using ChIP-seq, Logan, et al. could identify RAMP1 as a novel NKX3.1 target gene. Analysis using Oncomine, a cancer-specific bioinformatics database, found RAMP1 to be significantly upregulated in prostate cancer when compared with other cancers including the breast, bladder, liver, lung and pancreatic (*P* = 6.36 × 10^−47^). RAMP1 knockdown in two different prostate cancer cell lines showed decreases in proliferation, colony formation and numbers of cells in S phase of the cell cycle. In vivo, it produced smaller tumour sizes and reduced proliferation of cells. Downstream analysis found that levels of phosphorylated ERK1/2 were also reduced, linking RAMP1 to the MAPK pathway [39].

## The Role of the Calcitonin Family in Bone Microenvironment

A comprehensive review by Cornish, et al. has detailed the extensive role of the calcitonin peptides and their receptors in the regulation of bone metabolism. This review highlights findings from the past 30 years that show how calcitonin itself acts by lowering calcium levels in the blood triggered by inhibition of bone resorption, and the anti-resorptive properties of this peptide led to the discovery of its therapeutic uses in diseases associated with high bone turnover. More detailed studies have found that CT, CGRP and amylin can inhibit osteoclast motility and cause quiescence through a cAMP-dependent mechanism [40, 41]. However, CT is unique in its ability to alter intracellular calcium leading to osteoclast retraction, and this is believed to be achieved through cAMP-PKA mediated pathways that result in the disruption of cytoskeletal organization and alterations to cellular polarity of osteoclasts [42, 43]. CT can also act dose-dependently on mouse bone marrow cultures to induce the formation of multinucleated TRAP-positive cells at a much higher potency than CGRP and amylin [1].

Adrenomedullin has not been found to play a role in inducing hypocalcemia or inhibiting osteoclast function in normal physiology. Nevertheless, it does have a role to play in osteoblast activity and bone formation. Proliferation of osteoblasts in vitro can be stimulated by AM, CGRP and amylin, and in vivo studies have shown this can trigger bone formation. AM has been described previously as a potent osteoblast mitogen, due to its ability to increase proliferation at physiological concentrations of 10^−12^ M. AM and amylin at equipotency can dramatically increase bone formation and mineralized bone area, approximately threefold when injected daily into the hemicalvariae of adult mice [44]. Other in vivo models have found AM to have a profound effect on bone mass, increasing mouse tibial cortical width and trabecular bone volume by 21 and 45%, respectively. A significant increase in thickness of the epiphyseal growth plate was also found compared with control animals, leading to the conclusion that AM has a strong anabolic effect which may link to the normal regulation of bone mass [45].

## Prostate Cancer and the Bone Microenvironment: Adrenomedullin

An important mechanism through which tumour cells survive is their interaction with the bone microenvironment once metastasis has occurred. By signalling to the surrounding stroma, tumours are capable of progressing into a more aggressive phenotype. It is therefore apt to investigate whether the calcitonin family of peptides and their receptors play a role in influencing the bone microenvironment to promote tumourigenesis. Due to its potent effect on osteoblasts, AM could be capable of aiding tumour growth when metastasis to the bone has already occurred. To investigate this, Siclari et al. used a novel ex vivo model to co-culture breast cancer cells with mice calvarial bone. Tumour activity was then monitored by analysing changes in human tumour and mouse bone gene expression with qPCR. Tumour activity was significantly increased when treating with AM, and this was reversed with AM antagonists. Increases in expression of RANKL were seen in these co-cultures but it was found AM does not regulate this alone, which is consistent with data showing its lack of effect on osteoclasts. However, breast cancer cells co-cultured with the bone cause a decrease in bone RANKL and an increase in tumour cell RANKL expression. This effect is reversed by the treatment of AM, so RANKL levels are higher in bone than in tumour cells, this can be abolished using an AM antagonist. Previous studies of a vicious cycle in bone and breast cancer implicated PTHrP but expression of this peptide remained unchanged in all conditions leading to the conclusion that PTHrP is not responsible [46•].

Results of this investigation suggest that AM can increase tumour-induced osteolysis by altering localised production of RANKL, and that this change can be blocked using an AM antagonist. Nevertheless, there is much evidence that addition of AM to bone cultures does not stimulate osteoclast proliferation. The authors suggest that AM must be stimulating RANKL expression either by its effect on osteoblasts or by acting on tumour cells to release signals that then act on RANKL. They reason that AM has been shown to increase IL-6 expression in mesenchymal cells, but this has not been studied in bone or cancer models. This role of AM in RANKL expression and bone metastasis remains ongoing, and further studies in prostate cancer could reveal the mechanism behind this [46•].

## Prostate Cancer and the Bone Microenvironment: Calcitonin

Immunostaining of prostate cancer patient tissues shows significant increases in CT in high grade prostatic intraepithelial neoplasia and malignant acini of the prostate. However, in bone metastasis of prostate cancer, there is intense cytoplasmic staining of CT, suggesting that it is secreted by tumour cells after implanting in the bone, possibly to stimulate the bone microenvironment. Immunofluorescent staining of both CT and CTR shows only expression of either the ligand or receptor in stages 2 and 3 of prostate cancer but that CT-CTR co-expression is seen only in stage 4. This provides evidence that the CT-CTR axis has a paracrine effect earlier in prostate cancer but converts to an autocrine function after tumour metastasis to the bone. CT-CTR expression is also increased when comparing metastatic prostate tissue with localised sites [47]. This is consistent with previous data that shows CT-CTR expression acts to enhance prostate cancer growth and progression to metastasis [12].

## Prostate Cancer and the Bone Microenvironment: Calcitonin Gene-Related Peptide

The metastasis of prostate cancer to the bone can lead to bone pain in many patients, and this is a topic of much discussion in the literature. Studies investigating bone pain in prostate cancer also provide evidence of tumour cells acting on the surrounding stromal cells to influence the bone microenvironment. Canine prostate cancer cells were labelled with GFP before being injected into mouse femurs. An anti-CGRP antibody was then used to tag CGRP-positive sensory nerves. In control animals, nerves grow along the long axis of the bone, and as single fibres with a linear morphology however are compared with tumour-bearing mice, these fibres were grown in a disorganised manner with a thick fibre density and sprouting directly from prostate cancer cell colonies. These CGRP+ fibres also expressed TrkA, a receptor for Nerve Growth Factor (NGF) which promotes growth of new fibres in developing and adult animals. Interestingly, prostate cancer cells do not express NGF, suggesting that their implantation into the bone causes a tumour-associated effect on stromal cells. Further staining using DAPI for all nuclear cells and GFP for prostate cancer cells showed that up to 90% of the cells in prostate cancer colonies were stromal rather than tumour cells [48].

The prostate gland contains both autonomic and sensory neurons as well as neuroendocrine cells that secrete neuropeptides such as CGRP, and it has been reported previously that in prostate tumours, these cells often occur near highly proliferative cells. It has also been shown that RAMP1 knockdown in prostate cancer cells causes increases in MAPK, ERK1/2 and IL-6 [39]. Therefore, the presence of CGRP+ nerves in prostate cancer models indicates that secretion of CGRP can influence the bone microenvironment. Tumour-associated factors, like CGRP, can trigger the promotion of pathological sprouting of sensory neurons by increasing NGF expression.

## Conclusions

Findings over the past 5 years have provided much evidence for the role of the calcitonin family of peptides in prostate cancer and aiding metastasis to the bone. Not only do they seem to exert their effect to promote tumorigenesis but are also able to alter the bone microenvironment to further prostate cancer progression. Although AM is known to have a potent effect on bone physiology, there could still be further study into how it can affect both bone and tumour cells during prostate cancer metastasis. There is a clear gap in the literature investigating how AM is interacting with the bone microenvironment either directly or indirectly. This information would be very useful when focusing on AM as a therapeutic target in prostate cancer, especially in more advanced stages of the disease. This would provide a precedent for the investigation of AM2 (Intermedin) which has already been linked with prostate cancer in clinical studies, and a further understanding of this peptide would be very beneficial in this field of research. RAMP1 has now also been implicated as a major driver in prostate cancer, and so, linking this to CGRP will hopefully prompt researchers to look at this peptide in more detail. There also exist gaps in the literature when assessing the role of calcitonin in prostate cancer. With recent studies providing more evidence, it is clear that calcitonin is another target that must be investigated. In addition, to successfully determine each of these peptides’ influence on prostate cancer and metastasis, it is imperative to consider their individual receptor components as targets themselves. It is therefore certain that future research into the calcitonin family of peptides and their receptors will eventually lead to identification of prognostic markers and potential therapeutic targets that will ultimately increase the life expectancy of prostate cancer patients dramatically.

## References

[1] Naot D, Cornish J (2008). The role of peptides and receptors of the calcitonin family in the regulation of bone metabolism. Bone.

[2] Kitamura K, Kangawa K, Kawamoto M, Ichiki Y, Nakamura S, Matsuo H (1993). Adrenomedullin: a novel hypotensive peptide isolated from human pheochromocytoma. Biochem Biophys Res Commun.

[3] Roh J, Chang CL, Bhalla A, Klein C, Hsu SYT (2004). Intermedin is a calcitonin/calcitonin gene-related peptide family peptide acting through the calcitonin receptor-like receptor/receptor activity-modifying protein receptor complexes. J Biol Chem.

[4] Christopoulos A, Christopoulos G, Morfis M, Udawela M, Laburthe M, Couvineau A (2003). Novel receptor partners and function of receptor activity-modifying proteins. J Biol Chem.

[5] Phelps E, Bezouglaia O, Tetradis S, Nervina JM (2005). Parathyroid hormone induces receptor activity modifying protein-3 (RAMP3) expression primarily via 3′,5′-cyclic adenosine monophosphate signaling in osteoblasts. Calcif Tissue Int.

[6] Zudaire E, Martinez A, Cuttitta F (2003). Adrenomedullin and cancer. Regul Pept.

[7] Hay DL, Walker CS, Poyner DR (2011). Adrenomedullin and calcitonin gene-related peptide receptors in endocrine-related cancers: opportunities and challenges. Endocr Relat Cancer.

[8] Cornish J, Naot D (2002). Amylin and adrenomedullin: novel regulators of bone growth. Curr Pharm Des.

[9] Cornish J, Callon KE, Bava U, Kamona SA, Cooper GJS, Reid IR (2001). Effects of calcitonin, amylin, and calcitonin gene-related peptide on osteoclast development. Bone.

[10] Ritchie CK, Thomas KG, Andrews LR, Tindall DJ, Fitzpatrick LA (1997). Effects of the calciotrophic peptides calcitonin and parathyroid hormone on prostate cancer growth and chemotaxis. Prostate.

[11] Sabbisetti VS, Chirugupati S, Thomas S, Vaidya KS, Reardon D, Chiriva-Internati M (2005). Calcitonin increases invasiveness of prostate cancer cells: role for cyclic AMP-dependent protein kinase A in calcitonin action. Int J Cancer.

[12] Shah GV, Thomas S, Muralidharan A, Liu Y, Hermonat PL, Williams J (2008). Calcitonin promotes in vivo metastasis of prostate cancer cells by altering cell signaling, adhesion, and inflammatory pathways. Endocr-Relat Cancer..

[13] Thomas S, Chigurupati S, Anbalagan M, Shah G (2006). Calcitonin increases tumorigenicity of prostate cancer cells: evidence for the role of protein kinase A and urokinase-type plasminogen receptor. Molecular endocrinology (Baltimore, Md).

[14] Ho CCK, Seong PK, Zainuddin ZM, Manaf MRA, Parameswaran M, Razack AHA (2013). Retrospective study of predictors of bone metastasis in prostate cancer cases. Asian Pac J Cancer P.

[15] Paget S (1989). The distribution of secondary growths in cancer of the breast. 1889. Cancer Metastasis Rev.

[16] Shah GV, Noble MJ, Austenfeld M, Weigel J, Deftos LJ, Mebust WK (1992). Presence of calcitonin-like immunoreactivity (iCT) in human prostate gland: evidence for iCT secretion by cultured prostate cells. Prostate.

[17] Oehler MK, Norbury C, Hague S, Rees MCP, Bicknell R (2001). Adrenomedullin inhibits hypoxic cell death by upregulation of Bcl-2 in endometrial cancer cells: a possible promotion mechanism for tumour growth. Oncogene.

[18] Pio R, Martinez A, Unsworth EJ, Kowalak JA, Bengoechea JA, Zipfel PF (2001). Complement factor H is a serum-binding protein for adrenomedullin, and the resulting complex modulates the bioactivities of both partners. J Biol Chem.

[19] Chirgwin JM, Mohammad KS, Guise TA (2004). Tumor-bone cellular interactions in skeletal metastases. J Musculoskelet Neuronal Interact.

[20] Smith RS, Gao L, Bledsoe G, Chao L, Chao JL (2009). Intermedin is a new angiogenic growth factor. Am J Physiol-Heart C.

[21] Zhang W, Wang LJ, Xiao F, Wei Y, Ke W, Xin HB (2012). Intermedin a novel regulator for vascular remodeling and tumor vessel normalization by regulating vascular endothelial-cadherin and extracellular signal-regulated kinase. Arterioscl Throm Vas.

[22] Hay DL, Walker CS, Poyner DR (2011). Adrenomedullin and calcitonin gene-related peptide receptors in endocrine-related cancers: opportunities and challenges. Endocr-Relat Cancer.

[23] Brand RE, Ding XZ, Young CM, Adrian TE (2002). The specificity of amylin for the diagnosis of pancreatic adenocarcinoma. International journal of gastrointestinal cancer.

[24] Gkika D, Prevarskaya N (2011). TRP channels in prostate cancer: the good, the bad and the ugly?. Asian J Androl.

[25] • Oulidi A, Bokhobza A, Gkika D, Vanden Abeele F, Lehen'kyi V, Ouafik L, et al. TRPV2 mediates adrenomedullin stimulation of prostate and urothelial cancer cell adhesion, migration and invasion. PLoS One. 2013;8(5):e64885. **This paper reveals a novel mechanism for adrenomedullin in promoting prostate cancer metastasis through TRPV2 calcium channels***.* 10.1371/journal.pone.0064885 PMC3669125 23741410

[26] Berenguer-Daize C, Boudouresque F, Bastide C, Tounsi A, Benyahia Z, Acunzo J (2013). Adrenomedullin blockade suppresses growth of human hormone-independent prostate tumor xenograft in mice. Clin Cancer Res: an official journal of the American Association for Cancer Research.

[27] Fernandez-Sauze S, Delfino C, Mabrouk K, Dussert C, Chinot O, Martin PM (2004). Effects of adrenomedullin on endothelial cells in the multistep process of angiogenesis: involvement of CRLR/RAMP2 and CRLR/RAMP3 receptors. Int J Cancer.

[28] Kaafarani I, Fernandez-Sauze S, Berenguer C, Chinot O, Delfino C, Dussert C (2009). Targeting adrenomedullin receptors with systemic delivery of neutralizing antibodies inhibits tumor angiogenesis and suppresses growth of human tumor xenografts in mice. FASEB journal : official publication of the Federation of American Societies for Experimental Biology..

[29] Oehler MK, Hague S, Rees MC, Bicknell R (2002). Adrenomedullin promotes formation of xenografted endometrial tumors by stimulation of autocrine growth and angiogenesis. Oncogene.

[30] Brekhman V, Lugassie J, Zaffryar-Eilot S, Sabo E, Kessler O, Smith V (2011). Receptor activity modifying protein-3 mediates the protumorigenic activity of lysyl oxidase-like protein-2. FASEB journal : official publication of the Federation of American Societies for Experimental Biology.

[31] Hikosaka T, Tsuruda T, Nagata S, Kuwasako K, Tsuchiya K, Hoshiko S (2011). Adrenomedullin production is increased in colorectal adenocarcinomas; its relation to matrix metalloproteinase-9. Peptides.

[32] Lu Y-M, Zhong J-B, Wang H-Y, Yu X-F, Li Z-Q (2015). The prognostic value of intermedin in patients with breast cancer. Dis Markers.

[33] Wang XL, Wang YY, He HD, Xie X, Yu ZJ, Pan YM (2015). Association of plasma intermedin levels with progression and metastasis in men after radical prostatectomy for localized prostatic cancer. Cancer Biomark.

[34] Thakkar A, Aljameeli A, Thomas S, Shah GV (2016). A-kinase anchoring protein 2 is required for calcitonin-mediated invasion of cancer cells. Endocr Relat Cancer.

[35] Aljameeli A, Thakkar A, Thomas S, Lakshmikanthan V, Iczkowski KA, Shah GV (2016). Calcitonin receptor-zonula Occludens-1 interaction is critical for calcitonin-stimulated prostate cancer metastasis. PLoS One.

[36] Disantagnese PA (1992). Neuroendocrine differentiation in carcinoma of the prostate—diagnostic, prognostic, and therapeutic implications. Cancer.

[37] Nagakawa O, Ogasawara M, Fujii H, Murakami K, Murata J, Fuse H (1998). Effect of prostatic neuropeptides on invasion and migration of PC-3 prostate cancer cells. Cancer Lett.

[38] Suzuki K, Kobayashi Y, Morita T (2009). Significance of serum calcitonin gene-related peptide levels in prostate cancer patients receiving hormonal therapy. Urol Int.

[39] Logan M, Anderson PD, Saab ST, Hameed O, Abdulkadir SA (2013). RAMP1 is a direct NKX3.1 target gene up-regulated in prostate cancer that promotes tumorigenesis. Am J Pathol.

[40] Alam ASMT, Moonga BS, Bevis PJR, Huang CLH, Zaidi M (1993). Amylin inhibits bone-resorption by a direct effect on the motility of rat osteoclasts. Exp Physiol.

[41] Owan I, Ibaraki K (1994). The role of calcitonin-gene-related peptide (CGRP) in macrophages—the presence of functional receptors and effects on proliferation and differentiation into osteoclast-like cells. Bone Miner.

[42] Yamamoto Y, Udagawa N, Okumura S, Mizoguchi T, Take I, Yamauchi H (2006). Effects of calcitonin on the function of human osteoclast-like cells formed from CD14-positive monocytes. Cell Mol Biol.

[43] Alam ASMT, Bax CMR, Shankar VS, Bax BE, Bevis PJR, Huang CLH (1993). Further-studies on the mode of action of calcitonin on isolated rat osteoclasts—pharmacological evidence for a 2nd site mediating intracellular Ca2+ mobilization and cell retraction. J Endocrinol.

[44] Cornish J, Reid IR (2001). Effects of amylin and adrenomedullin on the skeleton. J Musculoskelet Neuronal Interact.

[45] Cornish J, Callon KE, Bava U, Coy DH, Mulvey TB, Murray MA (2001). Systemic administration of adrenomedullin(27-52) increases bone volume and strength in male mice. J Endocrinol.

[46] Siclari VA, Mohammad KS, Tompkins DR, Davis H, McKenna CR, Peng X (2014). Tumor-expressed adrenomedullin accelerates breast cancer bone metastasis. Breast Cancer Res.

[47] Thakkar A, Bijnsdorp IV, Geldof AA, Shah GV (2013). Profiling of the calcitonin-calcitonin receptor axis in primary prostate cancer: clinical implications and molecular correlates. Oncol Rep.

[48] Jimenez-Andrade JM, Bloom AP, Stake JI, Mantyh WG, Taylor RN, Freeman KT (2010). Pathological sprouting of adult nociceptors in chronic prostate cancer-induced bone pain. J Neurosci.

